# Assembling Quality Genomes of Flax Fungal Pathogens from Oxford Nanopore Technologies Data

**DOI:** 10.3390/jof9030301

**Published:** 2023-02-26

**Authors:** Elizaveta A. Sigova, Elena N. Pushkova, Tatiana A. Rozhmina, Ludmila P. Kudryavtseva, Alexander A. Zhuchenko, Roman O. Novakovskiy, Daiana A. Zhernova, Liubov V. Povkhova, Anastasia A. Turba, Elena V. Borkhert, Nataliya V. Melnikova, Alexey A. Dmitriev, Ekaterina M. Dvorianinova

**Affiliations:** 1 Engelhardt Institute of Molecular Biology, Russian Academy of Sciences, Moscow 119991, Russia; 2 Moscow Institute of Physics and Technology, Moscow 141701, Russia; 3 Federal Research Center for Bast Fiber Crops, Torzhok 172002, Russia; 4 All-Russian Horticultural Institute for Breeding, Agrotechnology and Nursery, Moscow 115598, Russia; 5 Faculty of Biology, Lomonosov Moscow State University, Moscow 119234, Russia

**Keywords:** *Aureobasidium pullulans*, *Colletotrichum lini*, *Fusarium verticillioides*, *Fusarium moniliforme*, pathogens, flax, nanopore sequencing, genome assembly

## Abstract

Flax (*Linum usitatissimum* L.) is attacked by numerous devastating fungal pathogens, including *Colletotrichum lini*, *Aureobasidium pullulans*, and *Fusarium verticillioides* (*Fusarium moniliforme*). The effective control of flax diseases follows the paradigm of extensive molecular research on pathogenicity. However, such studies require quality genome sequences of the studied organisms. This article reports on the approaches to assembling a high-quality fungal genome from the Oxford Nanopore Technologies data. We sequenced the genomes of *C. lini*, *A. pullulans*, and *F. verticillioides* (*F. moniliforme*) and received different volumes of sequencing data: 1.7 Gb, 3.9 Gb, and 11.1 Gb, respectively. To obtain the optimal genome sequences, we studied the effect of input data quality and genome coverage on assembly statistics and tested the performance of different assembling and polishing software. For *C. lini*, the most contiguous and complete assembly was obtained by the Flye assembler and the Homopolish polisher. The genome coverage had more effect than data quality on assembly statistics, likely due to the relatively low amount of sequencing data obtained for *C. lini*. The final assembly was 53.4 Mb long and 96.4% complete (according to the glomerellales_odb10 BUSCO dataset), consisted of 42 contigs, and had an N50 of 4.4 Mb. For *A. pullulans* and *F. verticillioides* (*F. moniliforme*), the best assemblies were produced by Canu–Medaka and Canu–Homopolish, respectively. The final assembly of *A. pullulans* had a length of 29.5 Mb, 99.4% completeness (dothideomycetes_odb10), an N50 of 2.4 Mb and consisted of 32 contigs. *F. verticillioides* (*F. moniliforme*) assembly was 44.1 Mb long, 97.8% complete (hypocreales_odb10), consisted of 54 contigs, and had an N50 of 4.4 Mb. The obtained results can serve as a guideline for assembling a de novo genome of a fungus. In addition, our data can be used in genomic studies of fungal pathogens or plant–pathogen interactions and assist in the management of flax diseases.

## 1. Introduction

*Colletotrichum lini* Manns et Bolley, *Aureobasidium pullulans* (de Bary) Arnaud, and *Fusarium verticillioides* (Sacc.) Nirenberg (*Fusarium moniliforme* Sheldon) are the fungal flax (*Linum usitatissimum* L.) pathogens, which cause diseases leading to significant crop losses. Flax is a highly valued cultivated plant because of its broad use. It is used for manufacturing food additives for people and animals because of the lignans, omega-3, and high fiber content in flaxseed [1,2,3,4]. Flax oil is widely used in industry as a component of coatings and paints [5]. Flax fiber is a popular product for manufacturing cloth and paper [6]. Thus, economic profit from these valuable products depends on flax resistance to various pathogens, including *C. lini*, *A. pullulans*, and *F. verticillioides* (*F. moniliforme*) [7,8]. However, these types of pathogens demonstrate significant genetic diversity, which hinders the creation of universally resistant varieties [9,10,11,12,13].

To develop resistant varieties, it is essential to conduct detailed studies of phytopathogen genetics and determine genetic markers of pathogenicity. This knowledge is useful for phylogenetic studies and species resolution [14,15]. For initial identification, fungal barcodes can be used, e.g., the beta-tubulin (*TUB2*) gene or the ITS region [16,17,18,19]. However, molecular markers sometimes provide limited information on species. For example, in the *Aureobasidium* genus, using the ITS2 marker failed to distinguish between the species. In contrast, whole-genome phylogenetic analysis identified three separate species [20]. Thus, for the most complete representation of the pathogen at the genetic level, the sequence and structure of its genome should be revealed. For *C. lini*, the flax anthracnose causative agent, the genome sequence was unknown until this study.

For *A. pullulans*, full genome assemblies are available in the NCBI database. They have an average length of 28.5 Mb (23.8–31.0 Mb) and scaffold/contig level (https://www.ncbi.nlm.nih.gov/assembly/?term=aureobasidium%20pullulans, accessed on 10 January 2023). Nonetheless, obtaining complete genomes of this species is still reasonable due to the significant genetic diversity between the isolates. Sequencing 50 *A. pullulans* strains isolated from different sources revealed the absence of population structure. However, linkage disequilibrium analysis suggested high levels of recombination between *A. pullulans* strains [21]. This fact might explain the polyextremotolerance of the fungus and its adaptability to a variety of unfavorable conditions. Thus, the genome of a flax-isolated strain might become a useful source of information on markers of adaptation to *L. usitatissimum*.

For *F. verticillioides* (*F. moniliforme*), genome assemblies are also available in the NCBI database (https://www.ncbi.nlm.nih.gov/assembly/?term=Fusarium+verticillioides, accessed on 10 January 2023). The length of these assemblies is 42.7 Mb on average (41.8–44.7 Mb). Their levels vary from contig to chromosome. However, most of the genomes were assembled only from Illumina data and then scaffolded. As a result, they still have many gaps and low contig N50 values (<0.5 Mb). In addition, none of the sequenced *F. verticillioides* is specified as an isolate from flax. Meanwhile, *Fusarium* includes a broad range of remarkably diverse species [22,23,24,25]. Thus, an SSR marker analysis demonstrated that *F. verticillioides* isolates from maize are genetically diverse without correlation with a geographic region of isolation [26]. Therefore, sequencing the genome of an isolate from flax is beneficial for further research on the pathogenicity and evolution of the species.

In the current study, we performed sequencing on a third-generation sequencing platform. In comparison with second-generation sequencing, third-generation sequencing technologies (Pacific Biosciences (PacBio), Menlo Park, CA, USA and Oxford Nanopore Technologies (ONT), Oxford, UK) enable the construction of genomes with fewer gaps [27,28,29]. Notably, Oxford Nanopore Technologies allows the acquisition of super-long reads with a maximum length of 2.3 Mb (the maximum length obtained in a scientific laboratory [30]). However, the obtained volume of raw reads depends on many factors, such as the organism species, genome size and structure, and purity and mass of the extracted DNA [31,32,33]. Using an appropriate protocol for DNA extraction is a key factor in receiving a sufficient amount of raw data to obtain a high-quality whole-genome assembly [34,35,36,37,38]. Unfortunately, the accuracy of the obtained data is limited by the technology itself. Errors occur during the steps of sequencing and raw signal deciphering [39]. Nevertheless, basecalling parameters can still be varied to receive the optimal genome assembly.

Data accuracy and quantity are important factors in determining the quality of the resulting assembly [38,40,41]. Thus, our research aimed to study the effect of different types of ONT data (different coverage, accuracy, and species) on the quality of a genome assembly. We analyzed genome assemblers’ performance in relation to raw ONT read volume and basecalling quality threshold. The effectiveness of different polishing tools for ONT data was also tested on the optimal raw assemblies of the sequenced flax pathogens.

## 2. Materials and Methods

### 2.1. Fungal Material

The following strains were used from the collection of the Institute for Flax (Torzhok, Russia): highly pathogenic to flax *Colletotrichum lini* #811, highly pathogenic to flax *Aureobasidium pullulans* #16, and *Fusarium verticillioides* (*Fusarium moniliforme*) #366 with low pathogenicity to flax. Mycelium was cultivated in test tubes with potato dextrose agar.

### 2.2. DNA Extraction and Purification

Pure, high-molecular-weight DNA was obtained according to the previously developed protocol for *Fusarium oxysporum* f. sp. *lini*, with several modifications [42]. After the step of incubation with RNase A, DNA samples were left at 8 °C overnight. Then, DNA purification was continued the next day, according to the protocol. For *C. lini* #811, all the precipitated DNA was taken for library preparation. This resulted in relatively low data output. To receive more sequencing data for *A. pullulans* #16 and *F. verticillioides (F. moniliforme*) #366, we took about 3 g of fungal material instead of 1 g, and visible DNA precipitate was removed from the samples. Only the remaining DNA was further used. The quality and quantity of the extracted DNA were evaluated with spectrophotometry (NanoDrop 2000C spectrophotometer, Thermo Fisher Scientific, Waltham, MA, USA) and fluorometry (Qubit 4.0 fluorometer, Thermo Fisher Scientific, Waltham, MA, USA). For *A. pullulans* #16 and *F. verticillioides (F. moniliforme*) #366, DNA was additionally purified with AMPure XP beads (Beckman Coutler, Brea, CA, USA) to achieve higher purity.

### 2.3. DNA Library Preparation and Sequencing on the Oxford Nanopore Technologies Platform

SQK-LSK109 Ligation Sequencing Kit (Oxford Nanopore Technologies, Oxford, UK) was used to prepare libraries. Several modifications were introduced to the recommended manufacturer’s protocol: the time of incubation at the step of DNA recovery at 20 °C was increased from 5 to 20 min, and the time of ligation was increased from 10 to 60 min. The prepared libraries were sequenced on a MinION instrument with the FLO-MIN-106 R9.4.1 flow cell.

### 2.4. Genome Assembly

The obtained reads were basecalled using Guppy 6.0.1 and the dna_r9.4.1_450bps_sup.cfg config file with different quality filtration thresholds (min_qscore). For *C. lini* strain #811, min_qscore was taken in the range of 7 to 10. For *A. pullulans* #16 and *F. verticillioides* (*F. moniliforme*) #366, a default min_qscore of 10 was chosen. Porechop 0.2.4 was used to remove adapters. For each min_qscore value, draft assemblies were performed using Canu 2.2 (-nanopore-raw; -minInputCoverage = 5; -stopOnLowCoverage = 5; -genomeSize = 50 m (*C. lini* #811), -genomeSize = 30 m (*A. pullulans* #16), or -genomeSize = 45 m (*F. verticillioides* (*F. moniliforme*) #366)), Flye 2.8.1 (--genome-size 50000000 (*C. lini* #811), --genome-size 30000000 (*A. pullulans* #16), or --genome-size 45000000 (*F. verticillioides* (*F. moniliforme*) #366)), Miniasm 0.3-r179 (-x ava-ont), NextDenovo 2.5.0 (https://github.com/Nextomics/NextDenovo, accessed on 10 January 2023), Ra 0.2.1 (-x ont), Raven 1.5.1, Shasta 0.8.0 (Nanopore-Oct2021.conf), SmartDenovo, and Wtdbg-cns 1.1 (Wtdbg2 0.0) (-x ont; -g 50 m (*C. lini* #811), -g 30 m (*A. pullulans* #16), or -g 45 m (*F. verticillioides* (*F. moniliforme*) #366)) [43,44,45,46,47,48,49]. To analyze the quality of the obtained assemblies, BUSCO (Benchmarking Universal Single-Copy Orthologs) 5.3.2 and QUAST 5.0.2 were used [50,51]. The following datasets were used for the analysis with BUSCO: glomerellales_odb10 (*C. lini* #811), dothideomycetes_odb10 (*A. pullulans* #16), and hypocreales_odb10 (*F. verticillioides* (*F. moniliforme*) #366). The following reference genomes were used for evaluating assembly contiguity: *C. lini* #811—*Colletotrichum higginsianum* IMI 349063 (GCA_001672515.1, accessed on 10 January 2023, sequenced with PacBio, chromosome-level assembly); *A. pullulans* #16—*Aureobasidium pullulans* (GCA_903819485.1, sequenced with ONT, contig-level assembly); *F. verticillioides* (*F. moniliforme*) #366—*Fusarium verticillioides* (GCA_003316995.2, sequenced with Illumina, chromosome-level assembly). The obtained draft assemblies were polished with ONT reads (the same basecalling quality threshold as was used to assemble the genomes) with Homopolish 0.3.4 (specified option: -m R9.4.pkl), MarginPolish 1.3.0 (allParams.np.json) (https://github.com/UCSC-nanopore-cgl/MarginPolish, accessed on 10 January 2023), Medaka 1.5.0 (https://github.com/nanoporetech/medaka, accessed on 10 January 2023), NextPolish 1.4.0, Pepper 0.0.6, Racon 1.4.20 [52,53,54,55]. For Homopolish, databases for polishing were created from assemblies available in NCBI: for *C. lini* #811—*Colletotrichum higginsianum* IMI 349063 (GCA_001672515.1), *Colletotrichum fructicola* (GCA_009771025.1), *Colletotrichum scovillei* (GCA_011075155.1), *Colletotrichum australisinense* (GCA_014706365.1), *Colletotrichum echinochloae* (GCA_016618095.1), *Colletotrichum eleusines* (GCA_016807845.1), *Colletotrichum horii* (GCA_019693695.1), *Colletotrichum acutatum* (GCA_001593745.1), *Colletotrichum sansevieriae* (GCA_002749775.1), *Colletotrichum musae* (GCA_002814275.1); for *A. pullulans* #16—*Aureobasidium pullulans* (GCA_903819465.1, GCA_003574545.1, GCA_004917105.1, GCA_004917135.1, GCA_004917145.1, GCA_004917155.1, GCA_004917165.1, GCA_004917185.1, GCA_004917375.1, GCA_004917415.1); for *F. verticillioides* (*F. moniliforme*) #366—*F. verticillioides* (GCA_026119585.1, GCA_020882315.1, GCA_027571605.1, GCA_013759275.1, GCF_000149555.1, GCA_003316975.2, GCA_003316995.2, GCA_003317015.2, GCA_025503005.1, GCF_000149555.1). If required, all prior alignments before polishing were produced with Minimap2 [56].

To compare the final assemblies of *C. lini*, *A. pullulans*, and *F. verticillioides* (*F. moniliforme*) with available genomes of *Colletotrichum*, *Aureobasidium*, and *Fusarium* species, the following assemblies were analyzed using BUSCO (glomerellales_odb10) and QUAST: *Colletotrichum fructicola* (GCA_009771025.1), *Colletotrichum scovillei* (GCA_011075155.1), *Colletotrichum australisinense* (GCA_014706365.1), *Colletotrichum echinochloae* (GCA_016618095.1), *Colletotrichum eleusines* (GCA_016807845.1), *Colletotrichum horii* (GCA_019693695.1), *Colletotrichum acutatum* (GCA_001593745.1), *Colletotrichum sansevieriae* (GCA_002749775.1), *Colletotrichum musae* (GCA_002814275.1), *Aureobasidium pullulans* (GCA_903819465.1), *Aureobasidium pullulans* (GCA_003574545.1), *Aureobasidium zeae* (GCA_017580825.2), *Fusarium verticillioides* (GCA_026119585.1), *Fusarium verticillioides* (GCA_020882315.1), *Fusarium avenaceum* (GCA_025948275.1), *Fusarium verticillioides* (GCA_027571605.1), *Fusarium verticillioides* (GCA_013759275.1).

## 3. Results

### 3.1. Genome Assembly and Polishing

The purity of the sequenced DNA affects the volume and quality of raw nanopore reads. In this study, we used a previously developed protocol to extract pure high-molecular-weight DNA from the studied fungi [42]. For *C. lini* #811, the total DNA pool was used to prepare sequencing libraries, as no visible DNA precipitate could be isolated. However, this resulted in low data output. We received 1.65 Gb of raw ONT reads with an N50 of 15.7 kb (33× genome coverage for an expected genome length of 50 Mb). Most likely, long DNA fragments in the pool were insufficiently purified, resulting in a short lifetime of sequencing pores. We assumed that the isolation failure was due to a low amount of the input biological material. For *A. pullulans* #16 and *F. verticillioides* (*F. moniliforme*) #366, a greater mass of mycelium (2.5–3 g instead of 1 g) was taken for DNA isolation. Thus, visible DNA precipitate (likely presented by long but insufficiently pure DNA fragments) could be removed from the pool, and the remaining material was sequenced. We received the following volumes of raw ONT reads with the corresponding N50 parameters: 3.89 Gb (130× genome coverage for an expected genome length of 30 Mb) with N50 = 5.8 kb (*A. pullulans* #16) and 11.08 Gb (220× genome coverage for an expected genome length of 50 Mb) with N50 = 6.9 kb (*F. verticillioides* (*F. moniliforme*) #366).

**Figure 1:**
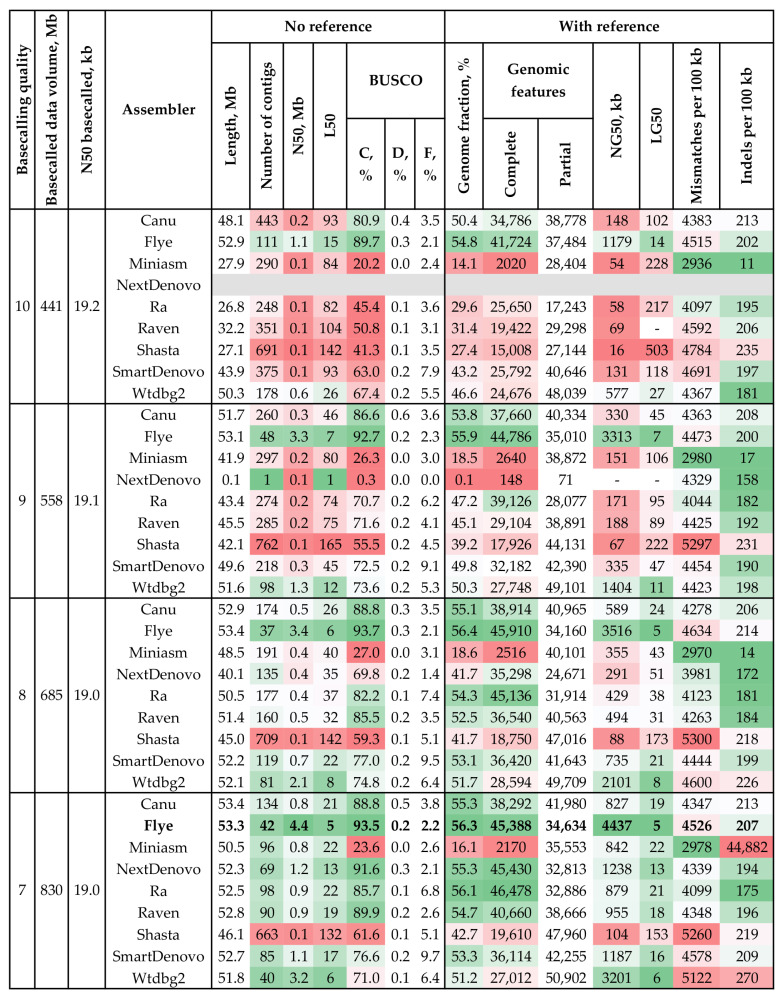
QUAST and BUSCO statistics of *C. lini* strain #811 draft genome assemblies. Basecalled data volume, millions of bases—data volume obtained after basecalling with different quality thresholds (min_qscore). N50 basecalled, kb—N50 of data obtained after basecalling with different quality thresholds (min_qscore). BUSCO: C—complete, D—duplicated, F—fragmented (the glomerellales_odb10 dataset). Reference—*Colletotrichum higginsianum* IMI 349063 (GCA_001672515.1). Genomic features: Partial—partially covered features (an assembly contains at least 100 bp of a feature (CDS, exons, etc.) but not its whole sequence). The used colors indicate estimations of the value quality from dark green (best) to bright red (worst). The grey line indicates the absence of an assembly. Bold font indicates the highest quality assembly.

For *C. lini* #811, the obtained sequencing data were basecalled with Guppy using the dna_r9.4.1_450bps_sup.cfg config file and different quality filtration thresholds (min_qscores of 7–10). The genome of the highly pathogenic *C. lini* #811 was assembled from these data with different tools (Figure 1, Table S1). We assessed the completeness and contiguity of the assembled sequences using BUSCO and QUAST. For each assembly, QUAST statistics were evaluated with and without a reference sequence. The contiguity of the assemblies was estimated by the number of contigs and the N50 and L50 parameters: the higher the N50 and the lower the number of contigs and L50, the more contiguous the assembly. Reference-based parameters were used to evaluate the completeness (reference genome fraction, identified reference genomic features (CDS, exons, etc.); higher values indicate higher completeness), contiguity (NG50, LG50), and accuracy (mismatches/indels per 100 kb; the lower these statistics, the more accurate the assembly) of the obtained assemblies. In the case of reference-based assessment, the compared query and reference genomes can be significantly different. Thus, it was not the QUAST statistics of an assembly that were of interest but their ratio to those of other obtained assemblies. BUSCO was used for evaluating assembly completeness. A higher fraction of complete benchmarking universal single-copy orthologs inherent to an analyzed species group indicated higher completeness of an assembly.

At all basecalling quality thresholds, the majority of the tools could assemble a genome of *C. lini* #811 with a completeness < 90% only. The assemblies with BUSCO completeness > 80% had an average length of 52.2 Mb (48.1–53.4 Mb), an average GC content of 54.04% (53.97–54.12%), and average duplication ratio values of 1.043 (1.037–1.048). For each min_qscore, the highest assembly completeness was achieved by Flye (up to 93.7%) and the lowest by Miniasm (less than 30%). However, NextDenovo demonstrated even worse BUSCO completeness (0.3%) than Miniasm at min_qscore = 9, as it failed to produce an assembly of a reasonable length. For min_qscore = 10, the assembler was unable to produce a consensus sequence. At high min_qscore values (9–10), all assemblers but Flye (at min_qscore = 9) failed to construct genomes with a BUSCO completeness of more than 90%. At high quality thresholds, the contiguity of the obtained assemblies was poor for most assemblers. Only Flye, at min_qscore = 9/10, and Wtdbg2, at min_qscore = 9, could obtain assemblies with N50 values of the megabase order and numbers of contigs fewer than 100. Generally, all assemblers demonstrated better results at the lower min_qscore values (7–8) than at the higher ones (9–10).

Notably, Flye outperformed other tools in terms of the key QUAST and BUSCO parameters. At each min_qscore, Flye produced assemblies with the best reference-based parameters, e.g., genome fraction and genomic features (more than 50% genome fraction for all min_qscore values). It obtained the most complete assembly from the basecalled data with min_qscore = 8: BUSCO completeness of 93.7%, 45,910 complete and 34,160 partial genomic features, and a genome fraction of 56.4%. At a min_qscore of 7, the tool produced the most contiguous assembly among all the raw genomes. It consisted of 42 contigs and had an N50 of 4.4 Mb. The second most continuous assembly was also performed by Flye at min_qscore = 8 (37 contigs, N50 = 3.4 Mb). Although BUSCO completeness at min_qscore = 8 (93.7%) was 0.2% higher than that at min_qscore = 7, the assembly at min_qscore = 7 had better QUAST statistics (N50, L50, and NG50). Therefore, we considered that the draft assembly at min_qscore = 7 is optimal in terms of contiguity and completeness.

**Figure 2:**
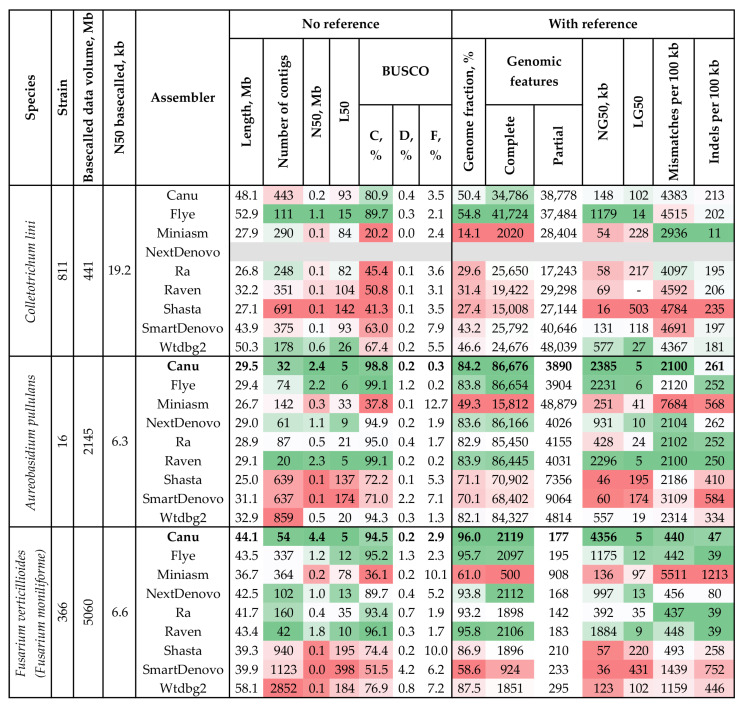
QUAST and BUSCO statistics of *C. lini* #811, *A. pullulans* #16, and *F. verticillioides* (*F. moniliforme*) #366 draft genome assemblies at min_qscore = 10. Basecalled data volume, millions of bases—data volume obtained after basecalling at min_qscore = 10. N50 basecalled, kb—N50 of data obtained after basecalling at min_qscore = 10. BUSCO: C—complete, D—duplicated, F—fragmented (the glomerellales_odb10 (*C. lini* #811), dothideomycetes_odb10 (*A. pullulans* #16), and hypocreales_odb10 (*F. verticillioides* (*F. moniliforme*) #366) datasets). Genomic features: Partial—partially covered features (if an assembly contains at least 100 bp of a feature (CDS, exons, etc.) but not its whole sequence). The used colors indicate estimations of the value quality from dark green (best) to bright red (worst). The grey line indicates the absence of an assembly. Bold font indicates the highest quality assemblies.

For *A. pullulans* #16 and *F. verticillioides* (*F. moniliforme*) #366, we obtained volumes of raw ONT data several times greater than those for *C. lini* #811. After basecalling with Guppy using the dna_r9.4.1_450bps_sup.cfg config file and default min_qscore = 10, the basecalled datasets were still several times bigger than those of *C. lini* #811 basecalled with min_qscore = 7 (2.15 Gb and 5.06 Gb vs. 0.83 Gb). Thus, taking into account the high genome coverage with ONT reads, we decided to use the data basecalled only with min_qscore = 10 for further genome assembly to reduce the number of low-quality reads. At min_qscore = 10, assemblers performed better for the larger datasets. Thus, the assemblies of *A. pullulans* #16 and *F. verticillioides* (*F. moniliforme*) #366 generally had better QUAST and BUSCO statistics than those of *C. lini* #811 (Figure 2, Tables S2 and S3). For *A. pullulans* #16, the assemblies by Flye and Raven had a BUSCO completeness of 99.1%, which was 0.3% more than the parameter for the Canu assembly. However, the assembly by Canu had the highest N50, reference genome fraction, genomic features, and NG50. Thus, the assembly by Canu was considered optimal: 98.8% BUSCO completeness, length = 29.5 Mb, N50 = 2.4 Mb, and number of contigs = 32. For *F. verticillioides* (*F. moniliforme*) strain #366, the most contiguous assembly was also obtained using the Canu assembler: N50 = 4.4 Mb against 1.8 Mb for Raven and 1.2 Mb for Flye; L50 = 5 against 10 for Raven and 12 for Flye. Reference-based analysis showed that this genome assembly has the highest genome fraction, genomic features, and NG50 parameters. Raven assembled the most complete genome according to BUSCO (96.1% completeness). However, N50 and other QUAST parameters of this assembly were substantially lower than those of the assembly by Canu. Therefore, the assembly by Canu was considered optimal for *F. verticillioides* (*F. moniliforme*) #366: 94.5% BUSCO completeness, length = 44.1 Mb, N50 = 4.4 Mb, and number of contigs = 54. Its BUSCO completeness can be further improved by polishing.

**Figure 3:**
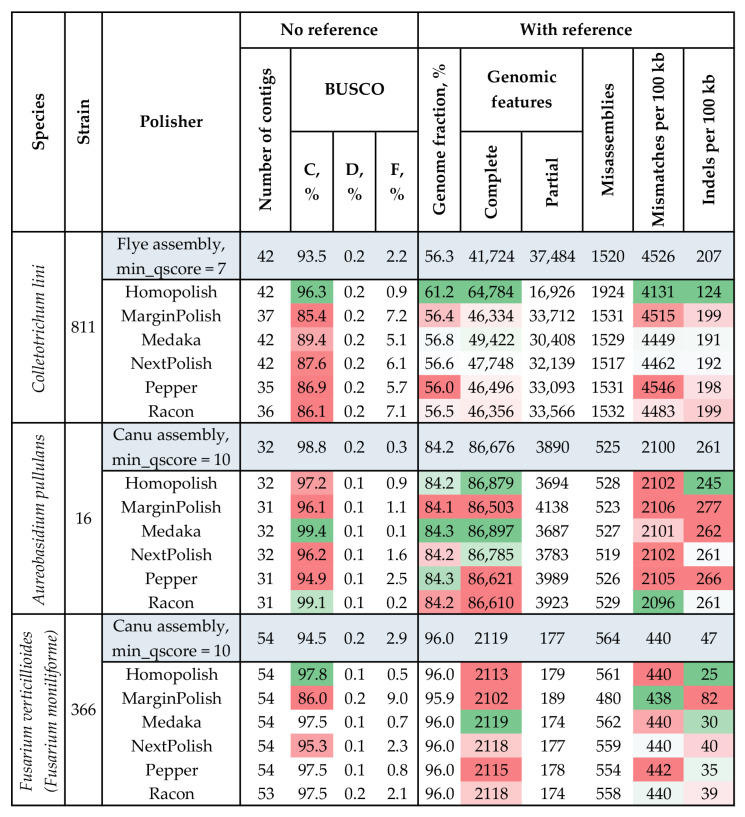
QUAST and BUSCO statistics of *C. lini* #811, *A. pullulans* #16, and *F. verticillioides* (*F. moniliforme*) #366 polished genome assemblies. BUSCO: C—complete, D—duplicated, F—fragmented (the glomerellales_odb10 (*C. lini* #811), dothideomycetes_odb10 *(A. pullulans* #16), and hypocreales_odb10 (*F. verticillioides* (*F. moniliforme*) #366) datasets). Genomic features: Partial—partially covered features (if an assembly contains at least 100 bp of a feature (CDS, exons, etc.) but not its whole sequence). The used colors indicate estimations of the value quality from dark green (best) to bright red (worst). Statistics in grey–blue are the ones of the best draft assembly that was further polished. All required prior alignments were made with Minimap2.

The best assemblies of the three strains (*C. lini* #811: Flye at min_qscore = 7, *A. pullulans* #16: Canu at min_qscore = 10, and *F. verticillioides* (*F. moniliforme*) #366: Canu at min_qscore = 10) were polished with ONT reads basecalled with the same basecalling quality threshold. Polishing was performed with six various tools. Each polisher was used in three iterations (Figure 3: first round of polishing; Tables S4–S6: all three rounds of polishing). In comparison with raw assemblies, polished ones should have better BUSCO completeness and contain more reference genome sequences and genomic features due to the improvement in sequence accuracy and reduction in the number of erroneous mismatches and indels. For *C. lini* #811, only Homopolish increased BUSCO completeness (from 93.5% to 96.3%). Genome fraction and genomic features also significantly rose after correction with Homopolish: from 56.30% to 61.15% and from 41,724 to 64,784, respectively. The tool significantly decreased mismatches and indels per 100 kb: from 4526 to 4131 and from 207 to 124, respectively. After the second round of polishing with Homopolish, these values were slightly refined: from 4131 to 4110 and from 124 to 116, respectively (Table S4). BUSCO completeness and reference genome fraction were also improved a little after the second polishing round. They rose from 96.3% to 96.4% and from 61.2% to 61.7%, respectively. The third round failed to make significant changes. Therefore, the assembly of the *C. lini* #811 genome after the second round of Homopolish was the most complete (BUSCO completeness = 96.4%) and contiguous (assembly length = 53.4 Mb, N50 = 4.4 Mb, 42 contigs, L50 = 5).

Polishing with Homopolish is based on the use of homologous sequences from the genomes of closely related species. Thus, a user needs to create a database of these sequences and pass it to the input of the polishing tool. We tried polishing the draft *C. lini* genome assembly using a single genome. To choose the closest sequence, we evaluated the QUAST parameters of the *C. lini* draft assembly using the genomes of *Colletotrichum* representatives: GCA_001672515.1, GCA_009771025.1, GCA_011075155.1, GCA_014706365.1, GCA_016618095.1, GCA_016807845.1, GCA_019693695.1, GCA_001593745.1, GCA_002749775.1, and GCA_002814275.1. *Colletotrichum higginsianum* GCA_001672515.1 had the highest reference genome fraction in the assembly of *C. lini* #811 and was used for polishing. However, the use of a single genome provided nearly the same results as when it was included in the database of several genomes. After each of the three polishing rounds, BUSCO completeness values and relative numbers of indels were equal to those received with a multi-genome database. After the first round of Homopolish, the number of complete reference genomic features was 18 more than for the assembly polished with a wider database. However, the second round of polishing resulted in the same value as that after polishing with several genomes. The relative number of mismatches also remained virtually unchanged compared to those for each polishing round with the multi-genome database. Thus, our previously obtained polished assembly could still be considered optimal.

For *A. pullulans* strain #16, polishing with only two tools led to an increase in BUSCO completeness: from 98.8% to 99.4% for Medaka and from 98.8% to 99.1% for Racon. The other polishers decreased the parameter. In addition, all tools except Medaka, Homopolish, and NextPolish lowered the number of complete reference genomic features. For most tools, changes in mismatches and indels per 100 kb were insignificant. The second round of polishing with Racon left BUSCO completeness unchanged (Table S5). The second round of polishing with Medaka failed to improve any parameters significantly: the genome fraction changed from 84.27% to 84.29%, indels per 100 kb improved from 262 to 259, and mismatches per 100 kb improved from 2101 to 2099. However, BUSCO completeness decreased from 99.4% to 97.1%. The third round of polishing with Racon significantly decreased BUSCO completeness from 99.1% to 96.4%. After the third round of polishing with Medaka, QUAST statistics changed insignificantly again: genomic features changed from 86,761 to 86,766, mismatches per 100 kb did not change, indels per 100 kb improved from 259 to 258, and BUSCO completeness remained the same. The second and third rounds of polishing with other tools had almost no effect on BUSCO completeness. Thus, polishing the assembly of *A. pullulans* #16 with one round of Medaka was optimal. The resulting assembly had 99.4% BUSCO completeness. It had a total length of 29.5 Mb, N50 of 2.4 Mb, 32 contigs, and L50 of 5.

One iteration of polishing the assembly of *F. verticillioides* (*F. moniliforme*) strain #366 with Homopolish, Medaka, Pepper, or Racon significantly increased BUSCO completeness. For Homopolish, the increase was the greatest: from 94.5% to 97.8%. Reference genome fraction and complete genomic features were not improved substantially by any tool. Homopolish greatly decreased the relative number of indels from 47 to 25. The second and third rounds of polishing with any tool resulted in insignificant changes (Table S6). For Homopolish, the second round improved indels per 100 kb only from 25 to 24. Meanwhile, other parameters remained nearly the same (mismatches per 100 kb, genomic features, and BUSCO completeness). The third round of Homopolish also failed to change most of these parameters. Thus, indels per 100 kb remained at 24. The second and third rounds of Medaka or Pepper made insignificant changes in the QUAST parameters. In contrast, BUSCO completeness decreased after the second and third rounds of Racon. Therefore, one round of polishing with Homopolish made the optimal assembly: BUSCO completeness = 97.8%, assembly length = 44.1 Mb, N50 = 4.4 Mb, 54 contigs, and L50 = 5.

### 3.2. Comparison with Available Genomes

To compare the obtained assemblies of *C. lini* #811, *A. pullulans* #16, and *F. verticillioides* (*F. moniliforme*) #366 with available assemblies from the NCBI database (accessed on 10 January 2023), genomes of the corresponding genus or species were downloaded and analyzed using BUSCO (the glomerellales_odb10 (*Colletotrichum*), dothideomycetes_odb10 (*Aureobasidium*), and hypocreales_odb10 (*Fusarium*) datasets) and QUAST. We analyzed the following assemblies obtained from ONT data: *Colletotrichum australisinense* (GCA_014706365.1) sequenced with ONT GridION; *Colletotrichum horii* (GCA_019693695.1) and *Colletotrichum scovillei* (GCA_011075155.1) sequenced with ONT PromethION; *Aureobasidium pullulans* (GCA_903819465.1) and *Fusarium avenaceum* (GCA_025948275.1) sequenced with ONT MinION.

In addition, we calculated statistics for hybrid assemblies from ONT and Illumina data: *Colletotrichum fructicola* (GCA_009771025.1), *Colletotrichum echinochloae* (GCA_016618095.1), *Colletotrichum eleusines* (GCA_016807845.1), *Fusarium verticillioides* (GCA_026119585.1), and *Fusarium verticillioides* (GCA_020882315.1).

**Figure 4:**
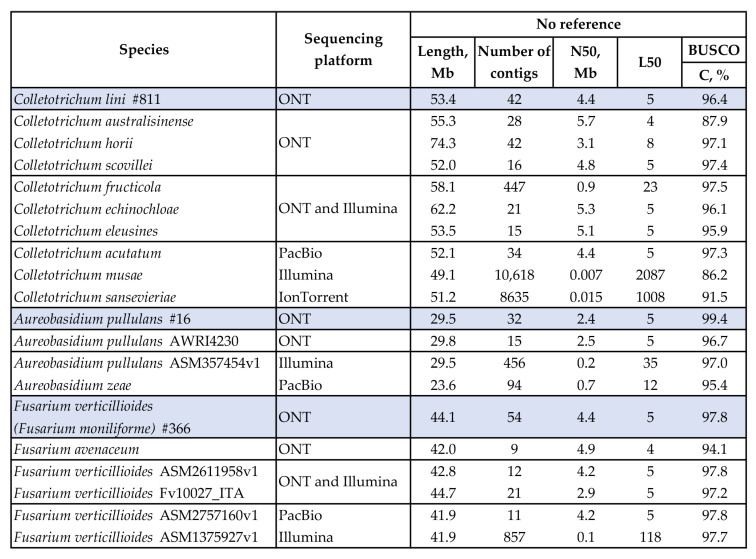
QUAST and BUSCO statistics of available genome assemblies of *Aureobasidium*, *Colletotrichum*, and *Fusarium* species. BUSCO: the dothideomycetes_odb10 (*Aureobasidium*), glomerellales_odb10 (*Colletotrichum*), and hypocreales_odb10 (*Fusarium*) datasets. The lines in grey–blue show statistics of the final assemblies of *C. lini* strain #811 (Flye, Homopolish x2), *A. pullulans* strain #16 (Canu, Medaka), and *F*. *verticillioides* (*F. moniliforme*) strain #366 (Canu, Homopolish).

To compare the assemblies with those constructed from data of sequencing technologies other than ONT, three genomes of *Colletotrichum* species (*Colletotrichum acutatum* (GCA_001593745.1) sequenced with PacBio, *Colletotrichum sansevieriae* (GCA_002749775.1) sequenced with IonTorrent, *Colletotrichum musae* (GCA_002814275.1) sequenced with Illumina); two genomes of *Aureobasidium* species (*Aureobasidium pullulans* (GCA_003574545.1) sequenced with Illumina, *Aureobasidium zeae* (GCA_017580825.2) sequenced with PacBio HiFi); and two genomes of *Fusarium verticillioides* species (*Fusarium verticillioides* (GCA_027571605.1) sequenced with PacBio HiFi, *Fusarium verticillioides* (GCA_013759275.1) sequenced with Illumina) were downloaded from the NCBI database (https://www.ncbi.nlm.nih.gov/, accessed on 10 January 2023) from all available data types (Figure 4).

BUSCO completeness of the available genomes varied from 86.2% to 97.8%. Among the assemblies from different data types, the highest average completeness was observed for the assemblies produced completely or partly from long reads. On average, BUSCO completeness was 95.5% for the assemblies only from long-read data (ONT and PacBio), 96.9% for the hybrid assemblies, and 93.1% for the short-read assemblies. Except for the *C. fructicola* and *A. zeae* assemblies, the analyzed long-read and hybrid assemblies consisted of 9–42 contigs and had an N50 in the megabase range. Although *C. fructicola* was sequenced on both ONT and Illumina platforms, assembly statistics took an intermediate position between those of the other assemblies from long-read data and short-read data. *A. zeae* was sequenced on the PacBio platform. However, the statistics of the assembly were not close to those of the other assemblies from long-read data. The assemblies from short reads had thousands of contigs, and their N50 were in order of kilobases. Thus, the contiguity and completeness of the assemblies obtained in this study were superior to those of the analyzed assemblies from short-read data. However, the assemblies of the flax pathogens had comparable characteristics to most of the analyzed assemblies from long reads.

## 4. Discussion

In this study, we sequenced three fungal strains pathogenic to flax—*C. lini*, *A. pullulans*, and *F. verticillioides* (*F. moniliforme*)—on the ONT platform. This long-read technology allowed us to assemble genomes with high QUAST and BUSCO parameters: megabase-order N50, dozens of contigs, and BUSCO completeness of more than 95%. However, if the assemblies were based on short reads, the number of contigs would be in the order of thousands, and the N50 values would be in the order of kilobases. For instance, the *C. sansevieriae* assembly from IonTorrent data has nearly nine thousand contigs and an N50 of 150 kb (GCA_002749775.1). Another example is the *C. musae* assembly from Illumina reads (GCA_002814275.1), which consists of ten thousand contigs and has an N50 of 7 kb.

Meanwhile, whole-genome analysis has the potential to reveal key virulence factors and provides the direction for a thorough study of fungal pathogenicity [57]. However, the studied genomes must possess enough contiguity and accuracy to guarantee confidence in the results of genomic analysis. A contiguous and complete genome of a pathogen will advance further molecular research on the species’ evolution, pathogenicity markers, genetic diversity, and plant–pathogen interactions [58,59,60,61,62,63,64]. This useful information can be retrieved from omics studies. Genome assemblies are used for mining genes, reconstructing phylogenetic relationships, studying recombination extents, revealing genetic determinants of adaptations, etc. [65,66,67]. Thus, constructing a quality assembly is a primary task in studying the genomics of fungal pathogens.

Assembling the optimal genome of an organism usually implies benchmarking at least several bioinformatics tools [68]. Although the approach can be time- and resource-consuming for large genomes, testing different software for bacteria and fungi is a feasible task [69,70,71]. First, draft genome assemblies can be produced with a variety of instruments [72,73]. Then, a researcher can choose an optimal software for polishing with genomic reads [73]. However, the performance of bioinformatics instruments depends on the given amount and quality of data, as well as the complexity and length of the studied genome [74,75].

In this study, to construct the optimal genome of *C. lini*, we tested the performance of different assembly and polishing tools provided with different amounts of data. Sequencing reads were basecalled with mean quality thresholds from 7 to 10 (the min_qscore parameter in Guppy). Thus, the obtained datasets could be classified into two groups: the smaller ones of higher quality and the larger ones of lower quality. For the data basecalled at the strictest threshold (min_qscore = 10), most assemblers produced genomes of low completeness and poor contiguity (Figure 1). Notably, NextDenovo failed to output any genome sequence at all. At min_qscore = 9, assemblers showed better QUAST and BUSCO statistics. However, only Flye assembled a genome with a BUSCO completeness greater than 90%. At min_qscore = 8, the quality of the obtained assemblies improved again. The lowest basecalling threshold resulted in the highest genome coverage (~17× per a 50-Mb genome), while the N50 of the basecalled data remained the same (19 kb at each min_qscore value). Thus, genome coverage was critical for assembly statistics. Lowering the basecalling threshold and increasing genome coverage ~1.9 times gradually improved the QUAST and BUSCO parameters of the assemblies, except for those by Wtdbg2 and SmartDenovo. The lowest basecalling threshold allowed us to receive the most contiguous and complete assembly.

At each min_qscore, Flye outperformed other assemblers in the main QUAST (genome length, number of contigs, N50) and BUSCO parameters. Even at ~9× genome coverage (min_qscore = 10), the assembly by Flye had an N50 of the megabase order. Meanwhile, several other assemblers demonstrated consistently poor results. Miniasm provided the lowest assembly completeness. The assemblies by Raven and Ra could reach only a kilobase-order N50. Canu, one of the most widely used tools, neither reached a completeness of >90% nor an N50 of >0.8 Mb at all basecalling thresholds. This fact can be explained by the differences between the implemented algorithms. Flye is based on a graphing approach, while Canu employs an overlap–layout–consensus (OLC) paradigm [43,44]. Therefore, low genome coverage might be insufficient for Canu to find high-confidence overlaps and construct contigs. Probably, altering assembler parameters could slightly improve the results. Nonetheless, Flye showed quality assembly statistics at the default parameters.

In addition to *C. lini*, we sequenced two more flax pathogen genomes. We obtained a greater data volume for *A. pullulans* and *F. verticillioides* (*F. moniliforme*) than for *C. lini*; the *A. pullulans* and *F. verticillioides* (*F. moniliforme*) genomes were covered with raw data ~130 and ~250 times, respectively. In comparison with the dataset for the *C. lini* #811 assembly, the datasets for *A. pullulans* #16 and *F. verticillioides* (*F. moniliforme*) #366 were larger. We assumed that data quality might not be critical for assembly accuracy at high data volumes. Therefore, we basecalled raw data with default parameters (min_qscore = 10). This reduced the genome coverage to ~70× and ~110×. Using the basecalled data, we tested the performance of different assembly software to choose the optimal draft genomes. For *A. pullulans* #16, Canu, Flye, NextDenovo, and Raven produced assemblies with the lowest number of contigs and highest N50. Although Flye and Raven assembled 0.3% more complete genome sequences than Canu, Canu still assembled a genome with a higher N50. In addition, the assembly by Canu had fewer contigs than that by Flye. For *F. verticillioides* (*F. moniliforme*) #366, assemblies by Canu and Raven had the best statistics. However, the assembly by Canu had a significantly higher N50 than the assemblies by other tools. Thus, for both *A. pullulans* and *F. verticillioides* (*F. moniliforme*), Canu constructed the most contiguous assemblies. At high genome coverage, this OLC assembler performed the best of all the tools.

In addition to high contiguity and completeness, accuracy is another important characteristic of a quality genome assembly. This parameter can be assessed using QUAST reference-based statistics and BUSCO completeness. Most representative QUAST statistics include the number of the identified reference genomic features (CDS, exons, etc.), mismatches, and indels per a certain number of base pairs. For *C. lini* #811, we calculated these statistics for the assemblies from basecalled data of different qualities. Despite different quality of genomic reads, we observed fluctuations in the relative number of indels for each assembler instead of a single tendency. For example, for Flye, the parameter changed in the following manner: 202–200–214–207 (min_qscores from 10 to 7). The relative number of mismatches showed the same trend for these assemblies. However, the number of the complete reference genomic features gradually increased with lowering data quality to min_qscore = 8. At min_qscore = 7, the statistic decreased, probably due to the poorer data quality. For the other assemblers, the parameters changed in varying ways. Therefore, the quality of the input data had an inconsistent effect. For *A. pullulans* #16 and *F. verticillioides* (*F. moniliforme*) #366, we applied a single basecalling threshold. Assemblies with the best BUSCO and basic QUAST statistics had the highest number of detected genomic features and one of the lowest numbers of mismatches and indels.

To improve the accuracy of the raw genomes, we performed polishing with ONT data of the same min_qscore values that were chosen as optimal for the high-quality draft assemblies. For *C. lini* #811 and *F. verticillioides* (*F. moniliforme*) #366, Homopolish polished raw assemblies to the smallest number of indels and the highest completeness and reference genome fraction. For *A. pullulans* #16, the polisher reduced BUSCO completeness. Since Homopolish corrects systematic errors using homologous sequences from the sequences provided by a user (database of 10 available *A. pullulans* assemblies was used for strain #16), it might have adjusted the *A. pullulans* genome to the provided sequences [53]. However, a large part of the *A. pullulans* genome can differ from those available in NCBI [21,76]. The highest assembly completeness of the fungal genome was achieved by Medaka. The parameter is crucial for an assembly, as it indicates the presence of the universal sequences of a taxon. Therefore, we regarded Medaka as the optimal polisher for *A. pullulans* strain #16.

Using Flye and Homopolish (two iterations), we received the first *C. lini* genome assembly from 1.65 Gb of raw ONT reads (N50 = 15.7 kb) basecalled at min_qscore = 7. The assembly is 53.4 Mb-long, consists of 42 contigs with an N50 of 4.4 Mb, and has a completeness of 96.4%. We compared the assemblies of the three sequenced phytopathogens with the available genomes of the corresponding genus or species (Figure 4). The completeness of the *C. lini* assembly is close to the median value of 96.6% for the deposited assemblies produced from ONT data, either completely or partially. The obtained assembly has an N50 higher than that of the genomes of *C. fructicola* (N50 = 0.9 Mb) and *C. horii* (N50 = 3.1 Mb). However, the other four genomes from ONT data (*C. scovillei*, *C. australisinense* (*nom. inval.*), *C. echinochloae*, and *C. eleusines*) have slightly higher N50 values (4.8–5.7 Mb) and a lower or the same number of contigs (15–42). Nonetheless, only the *C. australisinense* assembly has an L50 lower than that of the obtained *C. lini* assembly. The *C. lini* assembly has higher completeness and contiguity than assemblies from Illumina and IonTorrent data. However, these parameters are close to those of the *C. acutatum* assembly from PacBio reads. Therefore, the analyzed assemblies demonstrated comparable contiguity. Most likely, it was the relatively high N50 of the received sequencing reads that positively influenced the reached genome contiguity.

For *A. pullulans* and *F. verticillioides* (*F. moniliforme*), the optimal assemblies were obtained by Canu–Medaka (total length of 29.5 Mb, 32 contigs, an N50 of 2.4 Mb, 99.4% completeness) and Canu–Homopolish (total length of 44.1 Mb, 54 contigs, an N50 of 4.4 Mb, 97.8% completeness), respectively. The assembly of *A. pullulans* #16 has the highest completeness (99.4%) among all the analyzed assemblies of *Auereobasidium* representatives. This indicates that high coverage (more than 70×) with error-prone ONT reads results in improved accuracy of the resulting assembly. The N50 value of *A. pullulans* #16 is close to that of the *A. pullulans* AWRI4230 assembly from ONT data and higher than those of the assemblies from Illumina and PacBio reads. For *F. verticillioides* (*F. moniliforme*), the final assembly has a completeness (97.8%) equal to that of the *F. verticillioides* ASM2611958v1 genome from ONT reads and the *F. verticillioides* ASM2757160v1 genome from PacBio data. The assembly of *F. verticillioides* (*F. moniliforme*) #366 has an N50 slightly higher than the median value of 4.2 Mb for all the analyzed *Fusarium* genomes. Thus, the obtained amount of sequencing data for *A. pullulans* and *F. verticillioides* (*F. moniliforme*) (more than 70× and 110× genome coverage after basecalling, respectively) allowed us to construct assemblies with the main QUAST and BUSCO statistics close to those of the long-read genome assemblies from NCBI.

In this study, we assembled the genomes of the three flax pathogens (*C. lini*, *A. pullulans*, and *F. verticillioides* (*F. moniliforme*)) using ONT data and analyzed the influence of the basecalling read quality threshold and choice of assemblers and polishers on the assembly statistics. We defined the best approaches to obtain a genome assembly with the highest completeness and contiguity for each of the studied pathogens. As a result, high-quality assemblies of *C. lini* (53.37 Mb, N50 of 4.4 Mb, 96.4% complete), *A. pullulans* (29.5 Mb, N50 of 2.4 Mb, 99.4% complete), and *F. verticillioides* (*F. moniliforme*) (44.1 Mb long, N50 of 4.4 Mb, 97.8% complete) strains with known pathogenicity to flax were obtained for the first time.

## 5. Conclusions

Our results can guide the choice of a fungal de novo genome assembly strategy based on the use of ONT sequencing data. If low amounts of sequencing data were obtained (as in the case of *C. lini*), the genome coverage had more effect on assembly statistics than the quality of ONT reads. Therefore, using lower filtration threshold (min_qscore) values (7–8) for basecalling could be more effective than using the higher ones (9–10). Constructing the assemblies of *C. lini* demonstrated that Flye provided the best results at low genome coverage. Testing the assemblers’ performance at high genome coverage (the datasets for *A. pullulans* and *F. verticillioides* (*F. moniliforme*)) showed that Canu achieved the best results. Polishing with Homopolish yields better results on assemblies with low initial (before polishing) BUSCO completeness values (as in the case of *C. lini*). When the initial value of BUSCO completeness was already high (as for *A. pullulans*), Medaka and Racon were the most useful tools for increasing it.

The assembled genomes of the flax pathogens—*C. lini*, *A. pullulans*, and *F. verticillioides* (*F. moniliforme*)—with high completeness and contiguity can be included in comparative genomic studies of plant pathogens. For differently virulent strains of *C. lini*, *A. pullulans*, and *F. verticillioides* (*F. moniliforme*), such an analysis could be useful in determining pathogenicity mechanisms. Thus, our study contributes to future screening for genetic markers of pathogenicity and diagnosing or controlling fungal diseases of crops. However, in the present study, we obtained genome assemblies only for single representatives of the three examined species. Moreover, only few high-quality genome assemblies of these species with known virulence are available in public databases. Therefore, for studying the association of pathogenicity with genome features, it is necessary to obtain high-quality assemblies for larger sets of *C. lini*, *A. pullulans*, and *F. verticillioides* (*F. moniliforme*) with known virulence and, ideally, to create pan-genomes of these species.

## Data Availability

The generated dataset for this study can be found in the NCBI database under the BioProject accession numbers PRJNA929545, PRJNA929546, and PRJNA929547.

## Supplementary Materials

The following supporting information can be downloaded at: https://www.mdpi.com/article/10.3390/jof9030301/s1; Table S1: QUAST and BUSCO statistics of *Colletotrichum lini* strain #811 draft genome assemblies; Table S2: QUAST and BUSCO statistics of *Aureobasidium pullulans* strain #16 draft genome assemblies; Table S3: QUAST and BUSCO statistics of *Fusarium verticillioides* (*Fusarium moniliforme*) strain #366 draft genome assemblies; Table S4: QUAST and BUSCO statistics of *Colletotrichum lini* #811 polished genome assemblies; Table S5: QUAST and BUSCO statistics of *Aureobasidium pullulans* #16 polished genome assemblies; Table S6: QUAST and BUSCO statistics of *Fusarium verticillioides* (*Fusarium moniliforme*) #366 polished genome assemblies.
